# Supplementary material for: Sperm-inherited H3K27me3 impacts offspring transcription and development in C. elegans
Source: Nat Commun. 2019 Mar 20;10:1271. doi: 10.1038/s41467-019-09141-w (PMC6426959; doi:10.1038/s41467-019-09141-w)
Supplement: Supplementary file 1 — Supplementary Information [file 41467_2019_9141_MOESM1_ESM.pdf]

Sperm-inherited H3K27me3 impacts offspring transcription and development in *C. elegans*

Kaneshiro et al.

Supplementary Figure 1

Supplementary Figure 2

Supplementary Figure 3

Supplementary Figure 4

Supplementary Table 1

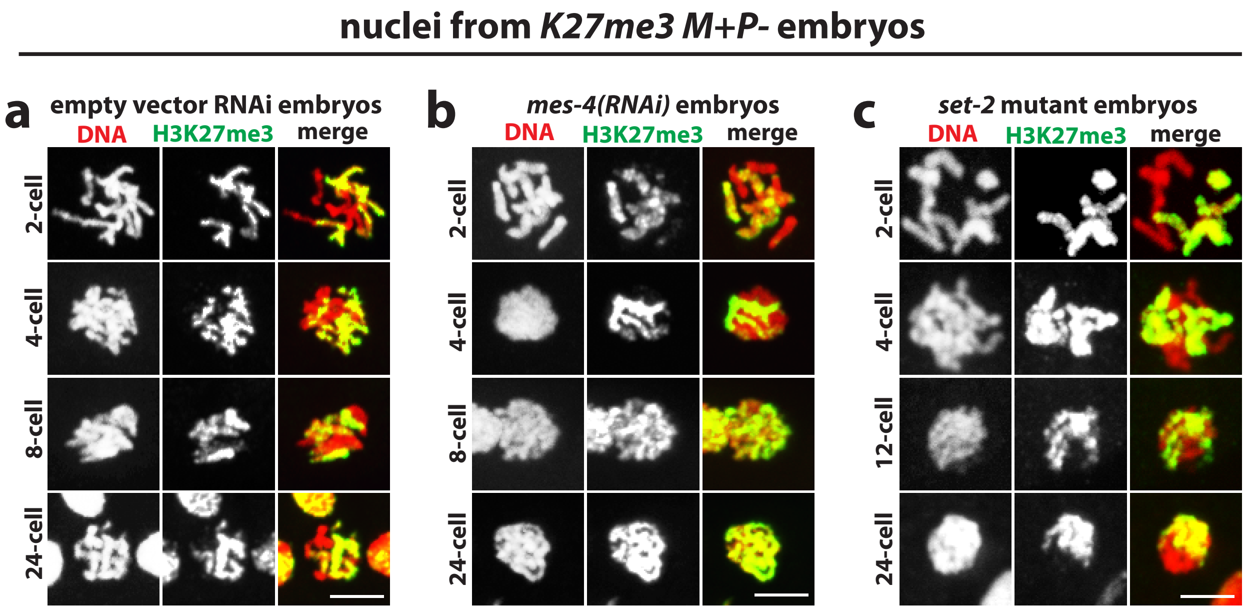

Supplementary Figure 1 Tracking the H3K27me3(-) state of sperm-inherited chromosomes in embryos depleted of the H3K36 methylator MES-4 or lacking the H3K4 methylator SET-2. Nuclei from *K27me3 M+P-* embryos from mothers treated with control RNAi (**a**), *mes-4* RNAi (**b**) or homozygous mutant for *set-2* (**c**). DAPI-stained DNA in red. H3K27me3 immunostaining in green. Scale bars represent 2µM.

***K27me3 M+P-* vs *M+P+* mRNA reads in germlines**

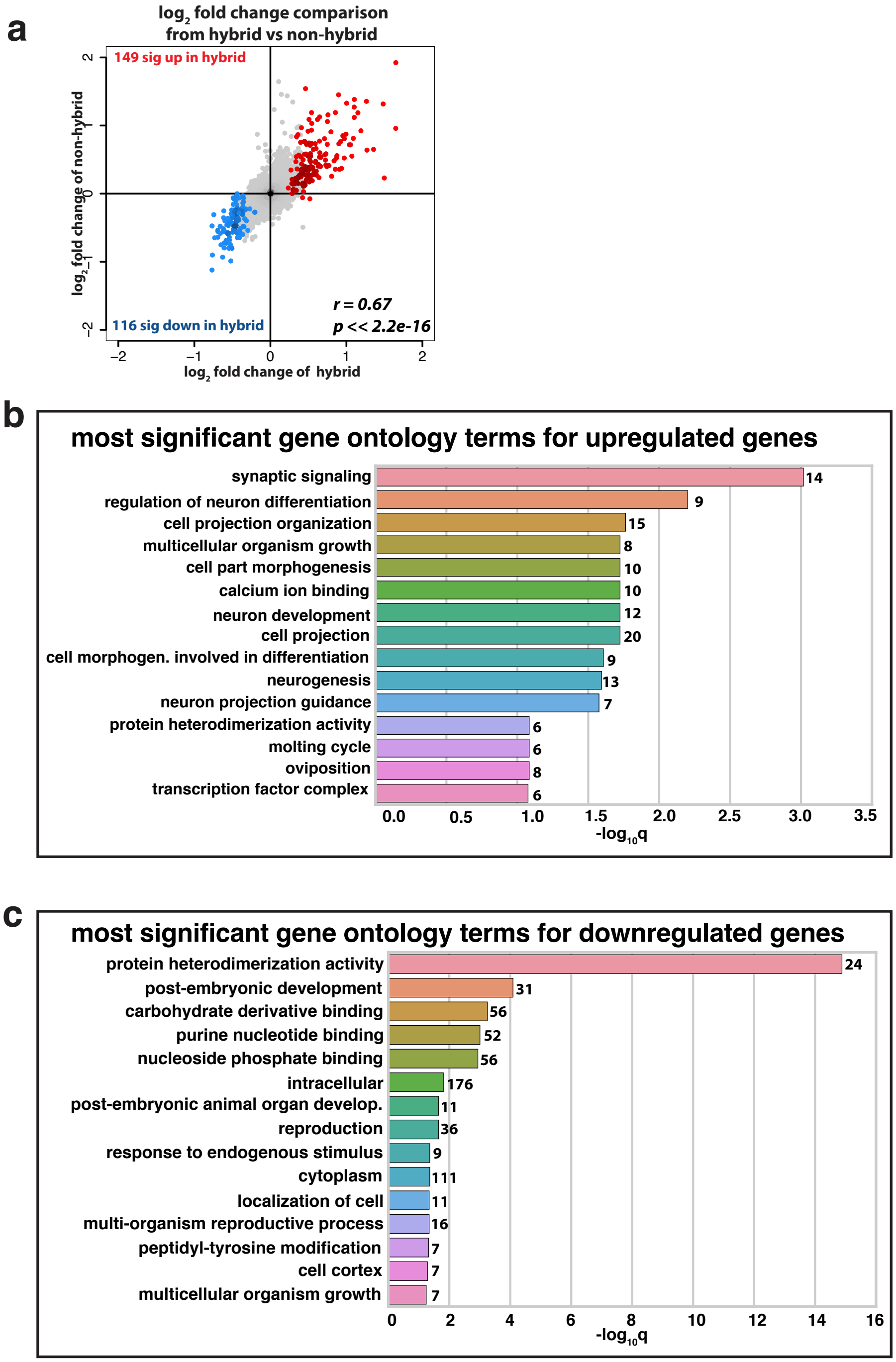

Supplementary Figure 2 Gene ontology analysis of significantly misregulated genes when sperm chromosomes are inherited lacking H3K27me3. **a** Log_2 fold change comparison of *K27me3 M+P-* vs *K27me3 M+P+* germline transcripts from hybrid worms (mother and father are from different wild-type isolates) versus non-hybrid worms (both parents are from the Bristol wild-type isolate). **b, c** Most significant gene ontology terms of upregulated genes (**b**) and downregulated genes (**c**) from the germlines of non-hybrid *K27me3 M+P-* vs *K27me3 M+P+* worms. Numbers of genes in each category are listed to the right of the histogram bins.

K27me3 M+P- vs *M+P+* mRNA reads in germlines

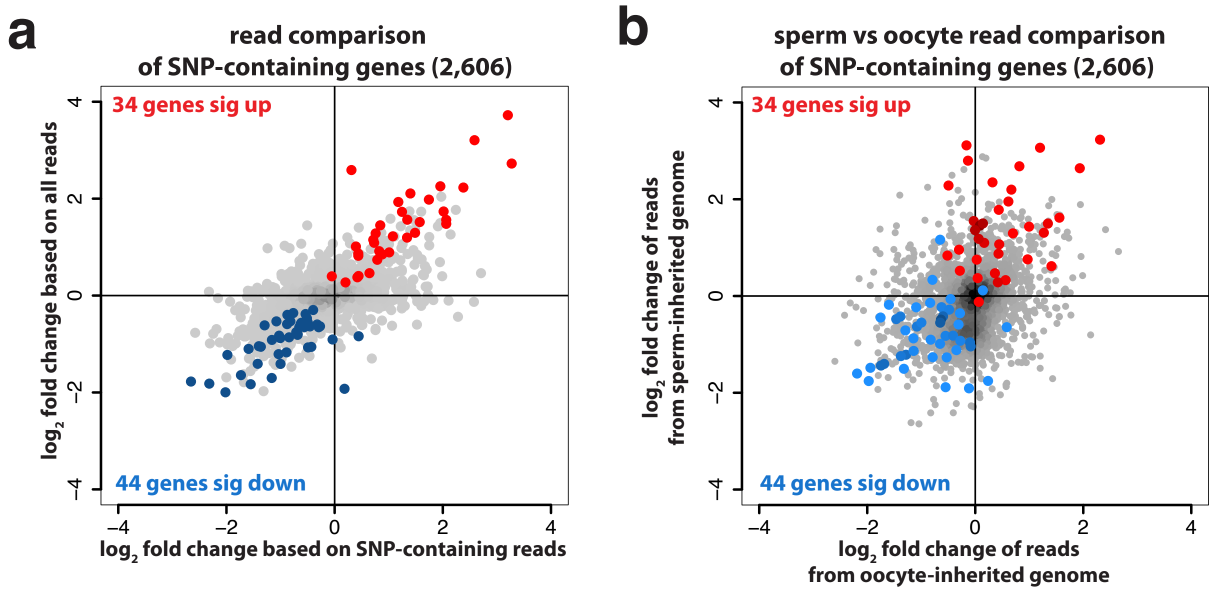

Supplementary Figure 3 Log_2 fold change comparisons of SNP-containing genes. Log_2 fold change comparison of *K27me3 M+P-* vs *K27me3 M+P+* germline transcripts for (**a**) SNP-containing reads (X-axis) vs all reads (Y-axis) and (**b**) reads that emanate from the oocyte-inherited (X-axis) vs sperm-inherited (Y-axis) genome. Genes that are significantly up- or downregulated ($p<0.1$) are highlighted in red and blue, respectively. Number of genes per category is shown in parentheses.

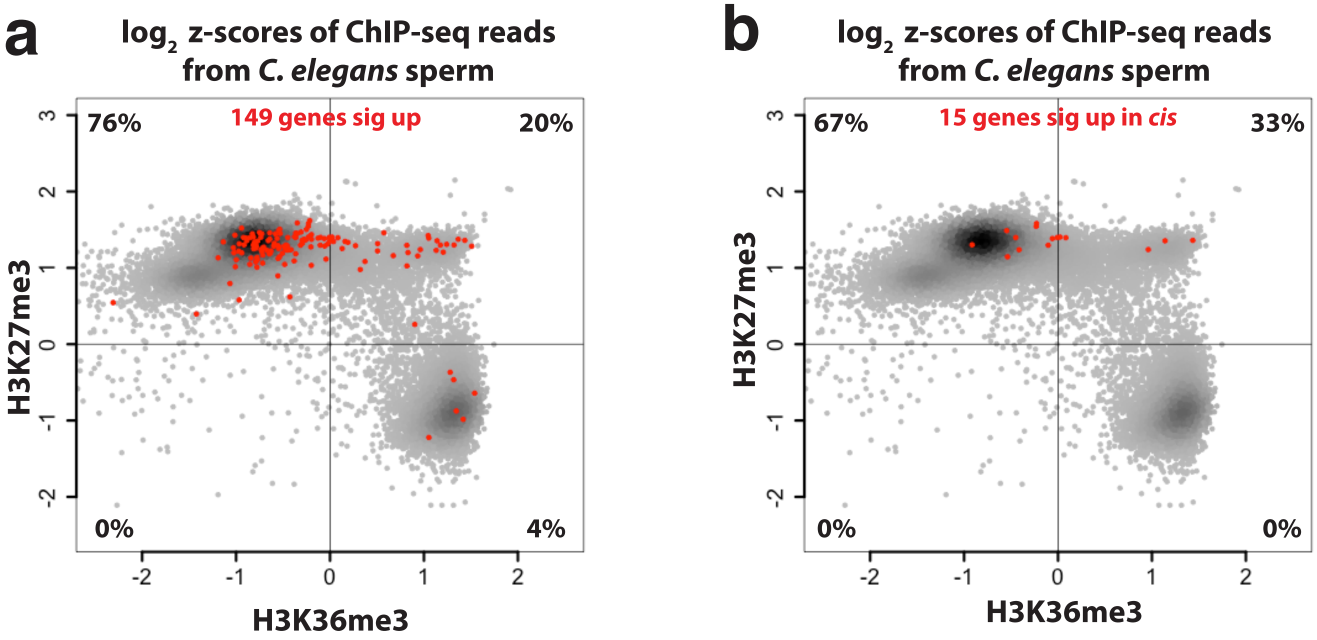

Supplementary Figure 4 Relative H3K27me3 versus H3K36me3 enrichment of upregulated genes in wildtype sperm. **a, b** Scatterplots of genes generated from published *C. elegans* sperm ChIP-seq experiments[10] highlighting genes that are significantly upregulated in *K27me3 M+P-* germlines. All genes that are significantly upregulated (FDR< 0.1) (**a**) and genes that are significantly upregulated in *cis* (dark red triangles in Fig. 2c) (**b**) are highlighted in red. Percent of highlighted genes that fall within each quadrant is indicated. Axes are mean normalized gene-body ChIP-seq signals.

worms analyzed		GFP+
M+P- L4 (n=146)		0.07%
M+P- sterile (n=267/894)	Day 1	16%
	Day 2	31%
M+P+ sterile (n=2/870)	Day 1	0%
	Day 2	0%

Supplementary Table 1 Percentage of worms expressing the neuronal marker *unc-119::gfp* in their germlines. M+P- and M+P+ indicate *K27me3 M+P-* and *K27me3 M+P+* genotypes. Number of worms scored is shown in parentheses. Day 1 and Day 2 of the same genotype represent the same worms scored on day 1 and then again on day 2 of adulthood.
