# Supplementary material for: Sperm-inherited H3K27me3 impacts offspring transcription and development in C. elegans
Source: Nat Commun. 2019 Mar 20;10:1271. doi: 10.1038/s41467-019-09141-w (PMC6426959; doi:10.1038/s41467-019-09141-w)
Supplement: Supplementary file 3 — Description of Additional Supplementary Files [file 41467_2019_9141_MOESM3_ESM.pdf]

Description of Additional Supplementary Files

File Name: Supplementary Data 1
Description: Table of SNP-containing genes used to assess oocyte or sperm allele specific changes in transcript reads from significantly misregulated genes (34 upregulated and 44 downregulated)
