# Supplementary material for: Sperm-inherited H3K27me3 impacts offspring transcription and development in C. elegans
Source: Nat Commun. 2019 Mar 20;10:1271. doi: 10.1038/s41467-019-09141-w (PMC6426959; doi:10.1038/s41467-019-09141-w)
Supplement: Supplementary file 5 — Reporting Summary [file 41467_2019_9141_MOESM5_ESM.pdf]

nature research

Corresponding author(s): *Double-blind peer review submissions: write DBPR and your manuscript number here instead of author names.*

Reporting Summary

Nature Research wishes to improve the reproducibility of the work that we publish. This form provides structure for consistency and transparency in reporting. For further information on Nature Research policies, see Authors & Referees and the Editorial Policy Checklist.

Statistical parameters

When statistical analyses are reported, confirm that the following items are present in the relevant location (e.g. figure legend, table legend, main text, or Methods section).

n/a	Confirmed	
☐	☒	The exact sample size (n) for each experimental group/condition, given as a discrete number and unit of measurement
☐	☒	An indication of whether measurements were taken from distinct samples or whether the same sample was measured repeatedly
☐	☒	The statistical test(s) used AND whether they are one- or two-sided<br>*Only common tests should be described solely by name; describe more complex techniques in the Methods section.*
☒	☐	A description of all covariates tested
☐	☒	A description of any assumptions or corrections, such as tests of normality and adjustment for multiple comparisons
☐	☒	A full description of the statistics including central tendency (e.g. means) or other basic estimates (e.g. regression coefficient) AND variation (e.g. standard deviation) or associated estimates of uncertainty (e.g. confidence intervals)
☐	☒	For null hypothesis testing, the test statistic (e.g. F, t, r) with confidence intervals, effect sizes, degrees of freedom and P value noted<br>*Give P values as exact values whenever suitable.*
☒	☐	For Bayesian analysis, information on the choice of priors and Markov chain Monte Carlo settings
☒	☐	For hierarchical and complex designs, identification of the appropriate level for tests and full reporting of outcomes
☐	☒	Estimates of effect sizes (e.g. Cohen's d, Pearson's r), indicating how they were calculated
☐	☒	Clearly defined error bars<br>*State explicitly what error bars represent (e.g. SD, SE, CI)*

Our web collection on statistics for biologists may be useful.

Software and code

Policy information about availability of computer code

Data collection	No software used
Data analysis	R version 3.5.1, DESeq2 version 1.22.1.

For manuscripts utilizing custom algorithms or software that are central to the research but not yet described in published literature, software must be made available to editors/reviewers upon request. We strongly encourage code deposition in a community repository (e.g. GitHub). See the Nature Research guidelines for submitting code & software for further information.

Data

Policy information about availability of data

All manuscripts must include a data availability statement. This statement should provide the following information, where applicable:

- Accession codes, unique identifiers, or web links for publicly available datasets
- A list of figures that have associated raw data
- A description of any restrictions on data availability

The datasets generated in this study are available at the Gene Expression Omnibus (GEO) repository, https://www.ncbi.nlm.nih.gov/geo/query/acc.cgi?acc=GSE123415.

Field-specific reporting

Please select the best fit for your research. If you are not sure, read the appropriate sections before making your selection.

☒ Life sciences ☐ Behavioural & social sciences ☐ Ecological, evolutionary & environmental sciences

For a reference copy of the document with all sections, see nature.com/authors/policies/ReportingSummary-flat.pdf

Life sciences study design

All studies must disclose on these points even when the disclosure is negative.

Sample size	4 biological replicates of next-generation sequencing experiments (RNA-seq) were performed. This sample size was based on experience and the standards in the field.
Data exclusions	No data were excluded.
Replication	Principal component analysis (PCA) of all replicates and genotypes showed appropriate clustering of samples. The results of sequencing and sterility analysis of hybrid worms were comparable to non-hybrid worms for each genotype analyzed.
Randomization	Experimental groups were defined by genotype.
Blinding	Blinding was not performed for sterility analysis as the phenotypes scored were obvious and experimenter influence is therefore negligable. Blinding was not performed for sequencing analysis as all samples were handled, processed, and analyzed identically.

Reporting for specific materials, systems and methods

Materials & experimental systems

n/a	Involved in the study	
☐	☒	Unique biological materials
☐	☒	Antibodies
☒	☐	Eukaryotic cell lines
☒	☐	Palaeontology
☐	☒	Animals and other organisms
☒	☐	Human research participants

Methods

n/a	Involved in the study	
☒	☐	ChIP-seq
☒	☐	Flow cytometry
☒	☐	MRI-based neuroimaging

Unique biological materials

Policy information about availability of materials

Obtaining unique materials	All materials, antibodies, and worm strains are available from the authors or from standard commercial and community sources.

Antibodies

Antibodies used	Kimura mouse anti-H3K27me3 MAb (MAb 1E7 clone CMA323, Wako cat# 309-95259), rabbit anti-H3K27me3 MAb (C36B11 Cell Signaling MAb#9733 lot#C36B11), mouse anti-GFP MAbs (Roche cat#11 814 460 001 lot#14158300), rabbit anti-UNC-64 serum, rabbit anti-PGL-1 serum, guinea pig anti-HTP-3 serum, Alexa Fluor: 488 goat anti-mouse (Life cat#A11001), 488 goat anti-rabbit (Molecular Probes cat#A-11008), 594 anti rabbit (Life cat#A11012), 594 goat anti-guinea pig (Molecular Probes cat#A11076)
Validation	Kimura mouse anti-H3K27me3 MAb (Egelhofer, T. A. et al. An assessment of histone-modification antibody quality. Nat. Struct. Mol. Biol. 18, 91–94 (2011)). In the Strome lab, staining is below detection in mes mutants that are incapable of generating H3K37me3.<br><br>Rabbit anti-H3K27me3 MAb (from manufacturer’s website: Tri-Methyl-Histone H3 (Lys27) (C36B11) Rabbit MAb detects endogenous levels of histone H3 only when tri-methylated on Lys27. The antibody does not cross-react with non-methylated, mono-methylated or di-methylated Lys27. In addition, the antibody does not cross-react with mono-methylated, di-methylated or tri-methylated histone H3 at Lys4, Lys9, Lys36 or Histone H4 at Lys20.) In the Strome lab, staining is below detection in mes mutants that are incapable of generating H3K37me3.

Mouse anti-GFP MAbs (manufacture's notes: Anti-GFP is a mixture of two high-affinity mouse monoclonal antibodies that were selected for their excellent performance in detection of GFP and a GFP fusion protein.) In the Strome lab, staining of GFP-tagged proteins with this antibody coincides with antibody staining of untagged endogenous proteins.
Rabbit anti-UNC-64 serum (Saifee, O., Wei, L. & Nonet, M. L. The Caenorhabditis elegans unc-64 locus encodes a syntaxin that interacts genetically with synaptobrevin. Mol. Biol. Cell 9, 1235–52 (1998))
Rabbit anti-PGL-1 serum (Kawasaki, I. et al. PGL-1, a predicted RNA-binding component of germ granules, is essential for fertility in C. elegans. Cell 94, 635–645 (1998))
Guinea pig anti-HTP-3 serum (MacQueen, A. J. et al. Chromosome sites play dual roles to establish homologous synapsis during meiosis in C. elegans. Cell 123, 1037–1050 (2005))

Animals and other organisms

Policy information about studies involving animals; ARRIVE guidelines recommended for reporting animal research

Laboratory animals	C. elegans hermaphrodites and males at all stages (as indicated in the manuscript) were used in this study.
Wild animals	The study did not involve wild animals.
Field-collected samples	The study did not involve samples collected from the field.
